# Association of initial lactate levels and red blood cell transfusion strategy with outcomes after severe trauma: a post hoc analysis of the RESTRIC trial

**DOI:** 10.1186/s13017-023-00530-7

**Published:** 2024-01-02

**Authors:** Yoshinori Kosaki, Takashi Hongo, Mineji Hayakawa, Daisuke Kudo, Shigeki Kushimoto, Takashi Tagami, Hiromichi Naito, Atsunori Nakao, Tetsuya Yumoto

**Affiliations:** 1 Department of Emergency, Critical Care, and Disaster Medicine, Faculty of Medicine, Dentistry, and Pharmaceutical Sciences, Okayama University, 2-5-1 Shikata-cho, Kita-ku, Okayama, 700-8558 Japan; 2 Department of Emergency Medicine, Hokkaido University Hospital, N14W5 Kita-ku, Sapporo, 060-8648 Japan; 3 Division of Emergency and Critical Care Medicine, Tohoku University Graduate School of Medicine, 1-1 Seiryo-Machi, Aoba-ku, Sendai, 980-8574 Japan; 4 Department of Emergency and Critical Care Medicine, Nippon Medical School Musashi Kosugi Hospital, 1-396 Kosugimachi, Nakahara-ku, Kawasaki, Kanagawa 211-8533 Japan

**Keywords:** Blood transfusion, Erythrocytes, Hemoglobin, Lactate, Trauma

## Abstract

**Background:**

The appropriateness of a restrictive transfusion strategy for those with active bleeding after traumatic injury remains uncertain. Given the association between tissue hypoxia and lactate levels, we hypothesized that the optimal transfusion strategy may differ based on lactate levels. This post hoc analysis of the RESTRIC trial sought to investigate the association between transfusion strategies and patient outcomes based on initial lactate levels.

**Methods:**

We performed a post hoc analysis of the RESTRIC trial, a cluster-randomized, crossover, non-inferiority multicenter trials, comparing a restrictive and liberal red blood cell transfusion strategy for adult trauma patients at risk of major bleeding. This was conducted during the initial phase of trauma resuscitation; from emergency department arrival up to 7 days after hospital admission or intensive care unit (ICU) discharge. Patients were grouped by lactate levels at emergency department arrival: low (< 2.5 mmol/L), middle (≥ 2.5 and < 4.0 mmol/L), and high (≥ 4.0 mmol/L). We compared 28 days mortality and ICU-free and ventilator-free days using multiple linear regression among groups.

**Results:**

Of the 422 RESTRIC trial participants, 396 were analyzed, with low (*n* = 131), middle (*n* = 113), and high (*n* = 152) lactate. Across all lactate groups, 28 days mortality was similar between strategies. However, in the low lactate group, the restrictive approach correlated with more ICU-free (*β* coefficient 3.16; 95% CI 0.45 to 5.86) and ventilator-free days (*β* coefficient 2.72; 95% CI 0.18 to 5.26) compared to the liberal strategy. These findings persisted even after excluding patients with severe traumatic brain injury.

**Conclusions:**

Our results suggest that restrictive transfusion strategy might not have a significant impact on 28-day survival rates, regardless of lactate levels. However, the liberal transfusion strategy may lead to shorter ICU- and ventilator-free days for patients with low initial blood lactate levels.

**Supplementary Information:**

The online version contains supplementary material available at 10.1186/s13017-023-00530-7.

## Background

Blood transfusions serve as a vital component in managing patients with trauma-related bleeding. Existing evidence suggests that conservative transfusion triggers are safe in critically ill patients [1–4]; however, these findings may not be applicable to those with active bleeding [5]. The prevailing guideline relevant to the management of major bleeding following trauma recommends targeting hemoglobin levels of 7 to 9 g/dL, based on previous studies that excluded patients with massive bleeding [6]. However, it is worth noting that this guideline was established without the support of high-quality evidence.

To address this gap, the Restrictive Transfusion Strategy for Critically Injured Patients (RESTRIC) trial was conducted to assess the non-inferiority of a restrictive red blood cell (RBC) transfusion strategy (hemoglobin level of 7 to 9 g/dL) compared to a liberal RBC transfusion strategy (hemoglobin level of 10 to 12 g/dL) in patients experiencing potential or ongoing massive hemorrhage after trauma during the acute phase of injury [7]. The trial showed that the two groups had similar 28-day survival rates and incidences of any type of adverse event.

Extensive literature has demonstrated that initial lactate values are independently associated with in-hospital mortality or the need for massive transfusion [8–11]. Given these findings, we hypothesized that the optimal hemoglobin target could vary according to initial lactate levels; a higher hemoglobin target may be preferable for patients with elevated initial lactate levels, whereas a lower target may be more appropriate for those with normal lactate levels to avoid potential harm from excessive transfusion.

To explore this, we conducted a post hoc analysis of patients enrolled in the RESTRIC trial, with the objective of examining the association between RBC transfusion strategy and patient outcomes based on initial lactate levels.

## Methods

### Study design

This was a post hoc analysis of the RESTRIC trial, a cluster-randomized, crossover, non-inferiority multicenter trial that was conducted across 22 institutions in Japan and involved 422 patients [7]. The design of the RESTRIC trial has been previously published [12]. In brief, the participating institutions were randomized to two schedules at a 1:1 ratio, comparing a restrictive RBC transfusion strategy (target hemoglobin level of 7 to 9 g/dL) with a liberal RBC transfusion strategy (target hemoglobin level of 10 to 12 g/dL) among adult patients with severe trauma at risk of major bleeding. The assigned transfusion strategy was implemented for all patients starting from the initial trauma resuscitation phase, upon arrival at the emergency department (ED), and continued until 7 days after hospital admission, intensive care unit (ICU) discharge, decision to withdraw active treatment, or death, whichever occurred first. Resuscitation with fluid or other blood components, such as fresh frozen plasma or platelets, was executed at the physician’s discretion. The protocol was approved by the ethics committees of each participating institution and that of the Japanese Association for the Surgery of Trauma, in line with the declaration of Helsinki. The present study was also approved by the Okayama University Hospital Ethics Committee (K2211-031).

### Participants

Inclusion criteria encompassed patients aged ≥ 20 years, experiencing one of the following, as judged by a physician: severe bleeding potentially leading to circulatory shock, suspected severe bleeding post-arrival at the ED, or potential for severe bleeding postoperatively during the acute trauma phase. From the participants registered in the RESTRIC trial, this post hoc analysis excluded those who withdrew consent, deviated from the transfusion strategy, were lost to follow-up, or had unavailable lactate level data upon ED arrival.

### Outcomes

The primary outcome was all-cause mortality at 28 days. Secondary outcomes included: time to death, cumulative RBC concentrate usage, ICU length of stay, and ventilator-, catecholamine-, and ICU-free days within the first 28 days. Patients who died within the first 28 days of arriving at the ED had zero days without events. Additionally, occurrence of organ (renal, hepatic, and respiratory) failure within the first 7 days and complications (including deep venous thrombosis, pulmonary embolism, cerebral infarction, myocardial infarction, bowel ischemia, transfusion-associated lung injury, and sepsis) within the first 28 days were assessed.

### Statistical analyses

We categorized patients into three subgroups based on lactate levels (mmol/L) upon ED arrival: low, middle, and high. This categorization is based on the findings of a previous study that also divided patients into three groups: low (< 2.5 mmol/L), middle (≥ 2.5 mmol/L and < 4.0 mmol/L), and high (≥ 4.0 mmol/L) [9]. Continuous data were expressed as medians with interquartile range (IQR), and categorical data as counts and percentages. Comparisons between the two groups employed the Mann–Whitney U test for continuous variables and the Chi-square test for categorical variables.

Cox regression analysis was utilized to estimate the hazard ratio (HR) and its 95% confidence interval (CI) for predicting 28 days mortality. Survival curves were generated using the Kaplan–Meier method and were compared with the log-rank test. Multiple linear regression analyses were employed to estimate adjusted effects on ICU-free, ventilator-free, and catecholamine-free days. The interaction *p* value, derived from the logistic linear regression model, was used to test the statistical significance of the interaction between lactate levels and transfusion strategy. Results were presented as β coefficients with 95% CI. Multiple logistic regression models were used to estimate the adjusted odds ratio (OR) and their 95% CI for the occurrence of any organ failure within 7 days and any complications within 28 days. These analyses were conducted for each lactate level category and were adjusted for factors including age, sex, systolic blood pressure on ED arrival, the presence of a severe head injury [defined as an Abbreviated Injury Scale (AIS) of 4 or 5 for the head], injury severity score (ISS), initial hemoglobin levels, and the need for major hemostatic interventions based on the previous studies [11, 13].

Additionally, in an exploratory subgroup analysis excluding patients with severe traumatic brain injury (head AIS of 4 or 5), similar methods were applied to estimate the HR for 28 days mortality and adjusted effects on ICU-free and ventilator-free days.

As a sensitivity analysis, we calculated the HR for 28 days mortality and the OR for the occurrence of organ failure within 7 days and complications within 28 days, using initial lactate levels as a continuous variable. For ICU-free and ventilator-free days, we estimated the *β* coefficients using linear regression. All these analyses were adjusted for the same variables.

All tests were two-tailed, and a *p* value of < 0.05 was deemed statistically significant. A threshold of *p* value for interaction was set at 0.10 [14]. Analyses were conducted using Stata SE version 17 statistical software (Stata-Corp LP, College Station, TX, USA) and Prism 10.0.3 (GraphPad, San Diego, CA).

## Results

**Fig. 1:**
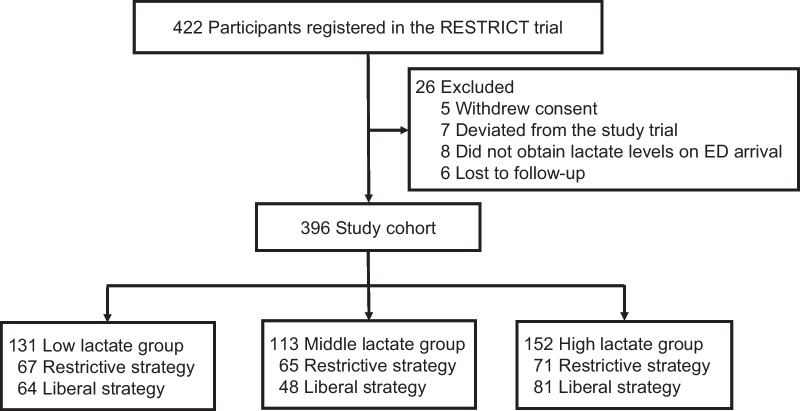
Patient selection flowchart

Of the 422 participants registered in the RESTRIC trial, 396 were included in the analysis (Fig. 1). Overall, the median age was 59 (IQR 44–74); 266 (67%) were male. The median lactate levels upon ED arrival were 3.2 (IQR 2.1 to 5.1) mmol/L. Based on lactate levels, participants were grouped into low lactate (< 2.5 mmol/L, n = 131), middle lactate (≥ 2.5 and < 4.0 mmol/L, *n* = 113), and high lactate (≥ 4.0 mmol/L, *n* = 152). The baseline demographics of the study groups are summarized in Table 1. Several imbalances were observed between the restrictive and liberal groups regarding initial hemoglobin levels and major hemostatic interventions in the low lactate group and platelet counts in the high lactate group. Trajectory of hemoglobin concentrations comparing between restrictive vs. liberal strategy group based on lactate levels is provided in Additional file 1.

**Table 1:** Patient characteristics based on initial lactate levels

	Low lactate (*n* = 131)			Middle lactate (*n* = 113)			High lactate (*n* = 152)		
	Restrictive (*n* = 67)	Liberal (*n* = 64)	*p* value	Restrictive (*n* = 65)	Liberal (*n* = 48)	*p* value	Restrictive (*n* = 71)	Liberal (*n* = 81)	*p* value
Age, median (IQR), y	66 (45–76)	63 (41–76)	0.85	64 (44–78)	62 (51–75)	0.65	53 (44–67)	51 (39–67)	0.33
Male sex, *n* (%)	45 (67)	36 (56)	0.20	41 (63)	35 (73)	0.27	51 (72)	58 (72)	0.98
*Comorbidities, n (%)*									
Chronic heart failure	2 (3)	2 (3)	0.96	2 (3)	0	0.22	0	0	N/A
Chronic respiratory failure	0	3 (5)	0.07	0	0	N/A	0	0	N/A
Chronic renal failure	1 (1)	0	0.33	0	0	N/A	0	1 (1)	0.41
Chronic liver failure	0	0	N/A	0	0	N/A	2 (3)	0	0.21
Immunosuppression	0	2 (3)	0.22	1 (2)	0	0.39	0	0	N/A
Types of injury, *n* (%)			0.31			0.86			0.73
Blunt	63 (94)	57 (89)		59 (91)	42 (88)		59 (83)	69 (85)	
Penetrating	4 (6)	7 (11)		5 (8)	5 (10)		12 (17)	12 (15)	
Blunt + penetrating	0 (0)	0 (0)		1 (2)	1 (2)		0	0	
*Physiologic status on arrival at emergency department*									
Respiratory rate, median (IQR), min	22 (18–24)	22 (18–27)	0.67	24 (19–30)	24 (18–30)	0.79	24 (20–30)	24 (20–30)	0.70
Heart rate, median (IQR), min	87 (74–107)	85 (75–96)	0.49	95 (78–120)	92 (75–105)	0.47	109 (86–130)	112 (85–131)	0.52
Systolic blood pressure, median (IQR), mmHg	124 (94–157)	122 (93–141)	0.51	106 (88–129)	112 (93–130)	0.61	85 (55–113)	96 (73–127)	0.07
Glasgow Coma Scale, median (IQR)	14 (13–15)	13 (12–15)	0.05	13 (11–15)	13 (8–14)	0.51	11 (6–14)	13 (7–14)	0.27
Injury severity score, median (IQR)	19 (13–26)	22 (14–27)	0.42	25 (14–36)	25 (15–32)	0.63	26 (16–41)	27 (17–35)	0.51
Severe traumatic brain injury, *n* (%)^a^	10 (15)	12 (19)	0.56	15 (23)	12 (25)	0.81	16 (23)	12 (15)	0.22
*Laboratory results on arrival at emergency department*									
Platelet, median (IQR), × 10^4^/μL	21.6 (18.3–25.3)	23.9 (17.4–28.3)	0.27	22.9 (18.3–28.9)	21.9 (18.7–25.1)	0.20	21.1 (15.9–25.1)	23.8 (18.4–28.6)	0.01
PT-INR, median (IQR)	1.02 (0.99–1.07)	1.04 (0.97–1.13)	0.82	1.09 (1.00–1.20)	1.04 (0.97–1.13)	0.10	1.14 (1.08–1.26)	1.12 (1.05–1.24)	0.49
Fibrinogen, median (IQR), mg/dL	236 (202–280)	234 (188–291)	0.88	221 (178–273)	204 (178–246)	0.16	185 (147–221)	185 (146–238)	0.56
Hemoglobin, median (IQR), mg/dL	13.0 (11.6–14.2)	12.3 (10.1–13.6)	0.03	11.8 (11.2–13.5)	12.7 (10.9–13.5)	0.74	11.9 (10.1–13.9)	11.6 (10.8–13.2)	0.80
Lactate, median (IQR), mmol/L	1.8 (1.4–2.2)	1.8 (1.4–2.1)	0.56	3.1 (2.8–3.5)	3.1 (2.8–3.4)	0.76	6.4 (4.7–8.5)	5.4 (4.7–8.3)	0.37
Major hemostatic interventions, n (%)^b^	23 (34)	35 (55)	0.02	37 (57)	30 (63)	0.55	48 (68)	63 (78)	0.16
Head	3 (4)	2 (3)	0.69	2 (3)	0	0.22	3 (4)	4 (5)	0.83
Chest	0	5 (8)	0.02	1 (2)	5 (10)	0.04	4 (6)	8 (10)	0.33
Abdomen	10 (15)	10 (16)	0.91	18 (28)	15 (31)	0.68	25 (35)	30 (37)	0.82
Pelvic fracture	5 (7)	15 (23)	0.01	13 (20)	5 (10)	0.17	10 (14)	16 (20)	0.35
Retroperitoneal hemorrhage	1 (1)	5 (8)	0.08	3 (5)	4 (8)	0.42	3 (4)	4 (5)	0.83
Extremities/neck	5 (7)	2 (3)	0.27	7 (11)	5 (10)	0.95	6 (8)	9 (11)	0.58
Others	0	0	N/A	1 (2)	1 (2)	0.83	4 (6)	3 (4)	0.57

^a^Defined as Abbreviated Injury Scale for the head of 4 or 5

^b^Includes interventions performed during the first 6 h after emergency department arrival

*IQR* interquartile range, *PT-INR* prothrombin time/international normalized ratio

Cumulative RBC transfusion volumes within 24 h and 7 days are illustrated in Additional file 2. Significantly higher RBC transfusion volumes were found in the low lactate group both within 24 h and 7 days, and within 24 h in the middle lactate group. The difference was especially pronounced in the low lactate group. However, there was no significant difference observed in the high lactate group for both time frames.

### Outcomes

Table 2 shows study outcomes between the restrictive and the liberal group based upon the lactate levels. There was no significant difference in 28-day survival between the restrictive and the liberal groups, regardless of the lactate levels. No differences were observed in the ICU length of stay, event-free days, organ failure rate, and complications rate between the restrictive group and liberal groups across all lactate levels. However, an exception was noted in the low lactate group, where the restrictive group had longer ventilator-free days compared to the liberal group.

**Table 2:** Comparison of the outcomes between restrictive and liberal strategy group based on initial lactate levels

	Low lactate (*n* = 131)			Middle lactate (*n* = 113)			High lactate (*n* = 152)		
	Restrictive (*n* = 67)	Liberal (*n* = 64)	*P* value	Restrictive (*n* = 65)	Liberal (*n* = 48)	*P* value	Restrictive (*n* = 71)	Liberal (*n* = 81)	*P* value
28-day survival, n (%)	65 (97)	60 (94)	0.37	62 (95)	44 (92)	0.42	60 (85)	72 (89)	0.43
Length of ICU stay, median (IQR), days	4 (3–8)	6 (3–15)	0.05	6 (3–14)	6 (3–15)	0.60	9 (3–17)	6 (3–12)	0.55
*Event-free days during the first 28 days, median (IQR)*									
ICU-free days	23 (18–24)	21 (9–24)	0.06	18 (12–24)	19 (9–23)	0.27	15 (2–21)	17 (3–24)	0.29
Ventilator-free days	28 (26–28)	26 (20–28)	0.003	25 (20–28)	23 (16–28)	0.42	21 (5–26)	23 (17–26)	0.38
Catecholamine-free days	28 (28–28)	28 (28–28)	0.74	28 (28–28)	28 (26–28)	0.77	28 (24–28)	28 (26–28)	0.2
Organ failure during the first 7 days, n (%)	5 (7)	4 (6)	0.78	10 (15)	3 (6)	0.13	12 (17)	15 (19)	0.30
Renal failure	2 (3)	1 (2)	0.59	3 (5)	1 (2)	0.47	2 (3)	8 (10)	0.08
Respiratory failure	4 (6)	4 (6)	0.95	9 (14)	3 (6)	0.2	10 (14)	10 (12)	0.8
Liver failure	0	0	N/A	1 (2)	1 (2)	0.83	1 (1)	2 (2)	0.64
Any complications during the first 28 days, n (%)	11 (16)	7 (11)	0.36	12 (18)	9 (19)	0.97	13 (18)	15 (19)	0.97
Deep venous thrombosis	9 (13)	5 (8)	0.30	9 (14)	6 (13)	0.84	6 (8)	5 (6)	0.59
Pulmonary embolism	1 (1)	0	0.33	0 (0)	2 (4)	0.1	4 (6)	0	0.03
Cerebral infarction	1 (1)	0	0.33	2 (3)	0	0.22	2 (3)	2 (2)	0.89
Acute myocardial infarction	0	0	N/A	0	0	N/A	0	0	N/A
Bowel ischemia	0	0	N/A	0	0	N/A	1 (1)	1 (1)	0.93
Transfusion-related acute lung injury	0	0	N/A	0	0	N/A	0	0	N/A
Sepsis	1 (1)	2 (3)	0.53	1 (2)	2 (4)	0.39	3 (4)	8 (10)	0.18

*ICU* intensive care unit, *IQR* interquartile range

**Fig. 2:**
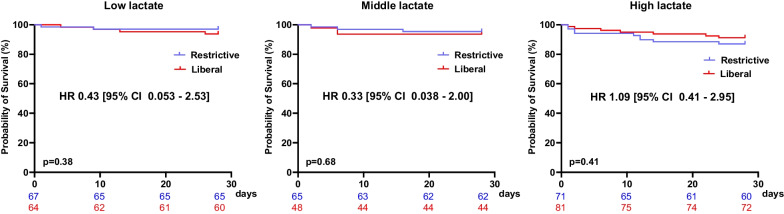
Kaplan–Meier survival analysis for 28-day survival comparing the restrictive group and the liberal group based on the initial lactate levels. HR is obtained via Cox regression analysis. Colored numbers at the bottom of the plot illustrate the number of patients at risk in the respective strategy at specified timepoints. *P* values were obtained with log-rank testing. *HR* hazard ratio

The log-rank test demonstrated that the high lactate group had higher 28 days mortality compared to low lactate group (Additional file 3). Regardless of lactate category, 28 days mortality was not different between the restrictive group and the liberal group as determined by the log-rank test. The HR for 28 days mortality in patients with liberal group when compared to the restrictive group was: HR 0.43 [95% CI 0.053 to 2.53] in the low lactate group; HR 0.33 [95% CI 0.038 to 2.00] in the middle lactate group; and HR 1.09 [95% CI 0.41 to 2.95] in the high lactate group (Fig. 2).

**Fig. 3:**
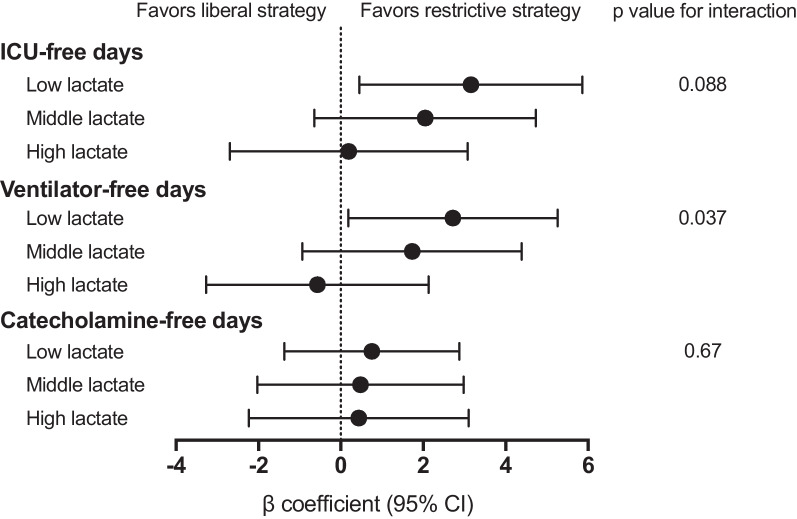
Multiple linear regression analyses to estimate adjusted effects of restrictive RBC transfusion strategy on ICU-free, ventilator-free, and catecholamine-free days, according to the initial lactate levels, setting liberal strategy as a reference. Analyses were conducted for each lactate level category and were adjusted for factors including age, sex, systolic blood pressure on ED arrival, the presence of a severe head injury (defined as an Abbreviated Injury Scale of 4 or 5 for the head), injury severity score, initial hemoglobin levels, and the need for major hemostatic interventions. Interactions between transfusion strategy and initial lactate levels were tested. *RBC* red blood cells, *ICU* intensive care unit, *ED* emergency department, *CI* confidence interval

Multiple linear regression analysis revealed that the restrictive strategy was associated with increased ICU-free days (*β* coefficient, 3.16; 95% CI 0.45 to 5.86) and ventilator-free days (*β* coefficient, 2.72; 95% CI 0.18 to 5.26) in the low lactate group, using the liberal strategy as a reference (Fig. 3). A significant interaction was observed between lactate levels and transfusion strategy for ventilator-free days (*p* for interaction = 0.088) and for ICU-free days (*p* for interaction = 0.37). Multiple logistic regression analysis demonstrated that restrictive strategy was not associated with any organ failure or complications, regardless of lactate levels (Additional file 4).

In an exploratory subgroup analysis excluding patients with severe traumatic brain injury, we could not determine the HR for the primary outcome due to an insufficient number of events within 28 days. However, the restrictive strategy continued to show an association with increased ICU-free days (*β* coefficient, 3.79; 95% CI 0.95 to 6.64) and ventilator-free days (*β* coefficient, 2.90; 95% CI 0.45 to 5.35) among the low lactate group (Additional file 5).

In the sensitivity analysis where initial lactate levels were treated as a continuous variable, the HR for 28 days mortality in patients with liberal group, when compared to the restrictive group was 0.90 [95% CI 0.43 to 1.88]. The restrictive strategy was associated with an increase in ICU-free days (*β* coefficient, 1.75; 95% CI 0.19 to 3.32), but there was no significant association with ventilator-free days (*β* coefficient, 1.03; 95% CI − 0.49 to 2.55). Finally, the restrictive strategy was not associated with an increased risk of organ failure (adjusted OR 1.12; 95% CI 0.57 to 2.22) or complications (adjusted OR 0.98; 95% CI 0.56 to 1.71).

## Discussion

In this post hoc analysis of the RESTRIC trial, we found no difference in 28-day survival between the restrictive and liberal RBC transfusion strategies during the acute phase following severe injury, irrespective of initial lactate levels. However, the restrictive strategy resulted in longer ICU-free and ventilator-free days in the low lactate group.

The findings from our post hoc analysis align with the primary outcomes of the RESTRIC trial, emphasizing the comparability of 28-day survival rates between the restrictive and liberal RBC transfusion strategies across varying lactate levels [7]. A predominant guideline on transfusion strategies for bleeding critically ill adults does not specify a target or threshold for hemoglobin levels after major trauma [15]. Recent updated European guidelines on management of major bleeding advocate for a target hemoglobin of 7 to 9 g/dL for RBC transfusion, consistent with previous recommendations [16].

While hyperlactatemia does not necessarily indicate the severity of tissue hypoxia, blood transfusion can improve tissue oxygenation [17], as hemoglobin plays a crucial role in delivering oxygen to tissues [18]. Of note, the correlation between tissue hypoxia and elevated lactate levels was pronounced during the initial resuscitation phase in critically ill patients [19]. Consequently, the guidelines emphasize that transfusion decisions should not be based solely on hemoglobin thresholds but should incorporate a more comprehensive assessment of the patient's clinical picture, including parameters such as lactate levels. Our study underscores this recommendation, as even though the 28-day survival rates demonstrated no significant differences, the restrictive strategy exhibited a slight tendency toward lower survival rates in the high lactate group, in contrast to the low and middle lactate groups.

The observation that the restrictive strategy was associated with increased ICU-free and ventilator-free days in the low lactate group is particularly noteworthy. This may be attributed to the fact that within the first 24 h and extending to 7 days, patients with low lactate levels receiving a liberal transfusion strategy were transfused more units of blood compared to the restrictive strategy. A prior meta-analysis concerning RBC transfusion in trauma patients revealed that increased RBC transfusion heightened the odds of experiencing multiple organ failure and acute lung injury [20]. Another study indicated that patients in the internal medicine ICU who underwent transfusion had extended ICU stays [21]. Although we do not have a clear explanation, there were no significant differences in complications or organ failure between the two strategies; however, there was a marked difference in ICU-free or ventilator-free days. Our study presents a critical finding that in patients with low lactate levels, a liberal transfusion strategy could be potentially harmful. This observation is particularly significant as it highlights the risks of excess transfusion in patients who may not require it. While lactate levels could reflect tissue perfusion and offer insights into the severity of hypoxia, our findings suggest that patients with low lactate levels might derive greater benefit from a restrictive transfusion approach. This is crucial, given the known risks and complications associated with blood transfusions. Excessive transfusion in these patients, who may not need it, could potentially lead to worse outcomes. In contrast, for patients with middle and high lactate levels, our analysis did not show any significant difference in ICU-free or ventilator-free days between the two strategies. It is conceivable that, in these patients, the potential benefits of improved oxygen delivery with a liberal transfusion strategy are counterbalanced by the risks associated with transfusion. Hence, our study underscores the importance of tailoring transfusion strategies based on individual patient characteristics such as lactate levels and cautions against the indiscriminate use of liberal transfusion practices, especially in patients without severe hypoxia or shock. These observations remained consistent even after excluding patients with severe traumatic brain injury, where initial increase in lactate levels is associated with unfavorable outcomes [22, 23].

There are several limitations in this study. First, the original RESTRIC study contained a significant error in sample size calculation, which was only identified after its completion. While the study was conducted with a sample size of 400 patients, a subsequent analysis revealed that the appropriate sample size should have been 6448 patients [12]. Second, our study has inherent limitations due to its post hoc nature. It is based on data from the RESTRIC trial, which was not originally designed to investigate the specific outcomes we analyzed. Therefore, potential confounding variables that were not accounted for in the original trial design, such as the precise time to definitive treatment, might influence our results. Third, while lactate levels provide valuable information about tissue perfusion, our analyses were based solely on a single initial time point. The dynamic changes in lactate levels, especially in response to interventions, might provide a more comprehensive understanding of the patient's status and could be an avenue for future research. Lastly, our analysis might lack the statistical power to discern differences in subgroups categorized by initial lactate levels.

Despite these limitations, the findings of our post hoc analysis offer valuable insights into the implications of RBC transfusion strategies based on lactate levels in trauma patients. It emphasizes the importance of individualized transfusion approaches and underscores the need to consider lactate levels as an integral aspect of this decision-making process. This finding also emphasizes the complexity of managing trauma patients and the need for a holistic approach that considers multiple clinical parameters, including lactate levels, hemodynamic stability, and the presence of other comorbidities.

## Conclusions

In our post hoc analysis of the RESTRIC trial, we found no significant difference in 28-day survival between restrictive and liberal RBC transfusion strategies after severe injury, regardless of initial lactate levels. However, our findings indicate a potential disadvantage associated with the liberal transfusion strategy, as evidenced by shorter ICU- and ventilator-free days in patients with low initial lactate levels. Further research is needed to validate these observations with more comprehensive clinical parameters.

### Supplementary Information

**Additional file 1**. Trajectory of hemoglobin concentrations in the restrictive versus liberal strategy groups according to initial lactate levels. Data are expressed as medians with interquartile ranges. Indicator of significance was reported by adjusted *P* value as *p < 0.05, **p < 0.01, and ***p < 0.001 in figure.

**Additional file 2**. Cumulative RBC transfusion volumes comparing between restrictive and liberal strategy based on initial lactate levels within 24 h and 7 days. Data are expressed as medians with interquartile ranges. Indicator of significance was reported by adjusted *P* value as *p < 0.05 and ****p < 0.0001 in figure. RBC: red blood cell.

**Additional file 3**. Kaplan–Meier survival analysis for 28-day survival comparing between the low, middle, and high lactate groups. Colored numbers at the bottom of the plot illustrate the number of patients at risk in the respective strategy at specified timepoints. *p = 0.041 compared with low the lactate group.

**Additional file 4**. Multiple logistic regression analyses to estimate adjusted effects of restrictive RBC transfusion strategy on any organ failure and any complications, according to the initial lactate levels, setting liberal strategy as a reference.

**Additional file 5**. Multiple linear regression analyses to estimate adjusted effects of restrictive vs. liberal RBC transfusion strategy on ICU-free and ventilator-free days after excluding patients with severe traumatic brain injury, according to the initial lactate levels. Analyses were conducted for each lactate level category and were adjusted for factors including age, sex, systolic blood pressure, injury severity score, initial hemoglobin levels, and the need for major hemostatic interventions. *RBC* red blood cells, *ICU* intensive care unit, *CI* confidence intervals.

## Data Availability

The datasets from this study are available from the corresponding author upon reasonable request.

## Abbreviations

AIS: Abbreviated injury scale
CI: Confidence intervals
ED: Emergency department
HR: Hazard ratio
ICU: Intensive care unit
ISS: Injury severity score
IQR: Interquartile range
OR: Odds ratio
RBC: Red blood cell
