# Supplementary figures and images for: Association of initial lactate levels and red blood cell transfusion strategy with outcomes after severe trauma: a post hoc analysis of the RESTRIC trial
Source: World J Emerg Surg. 2024 Jan 2;19:1. doi: 10.1186/s13017-023-00530-7 (PMC10763143; doi:10.1186/s13017-023-00530-7)

## Slide 1
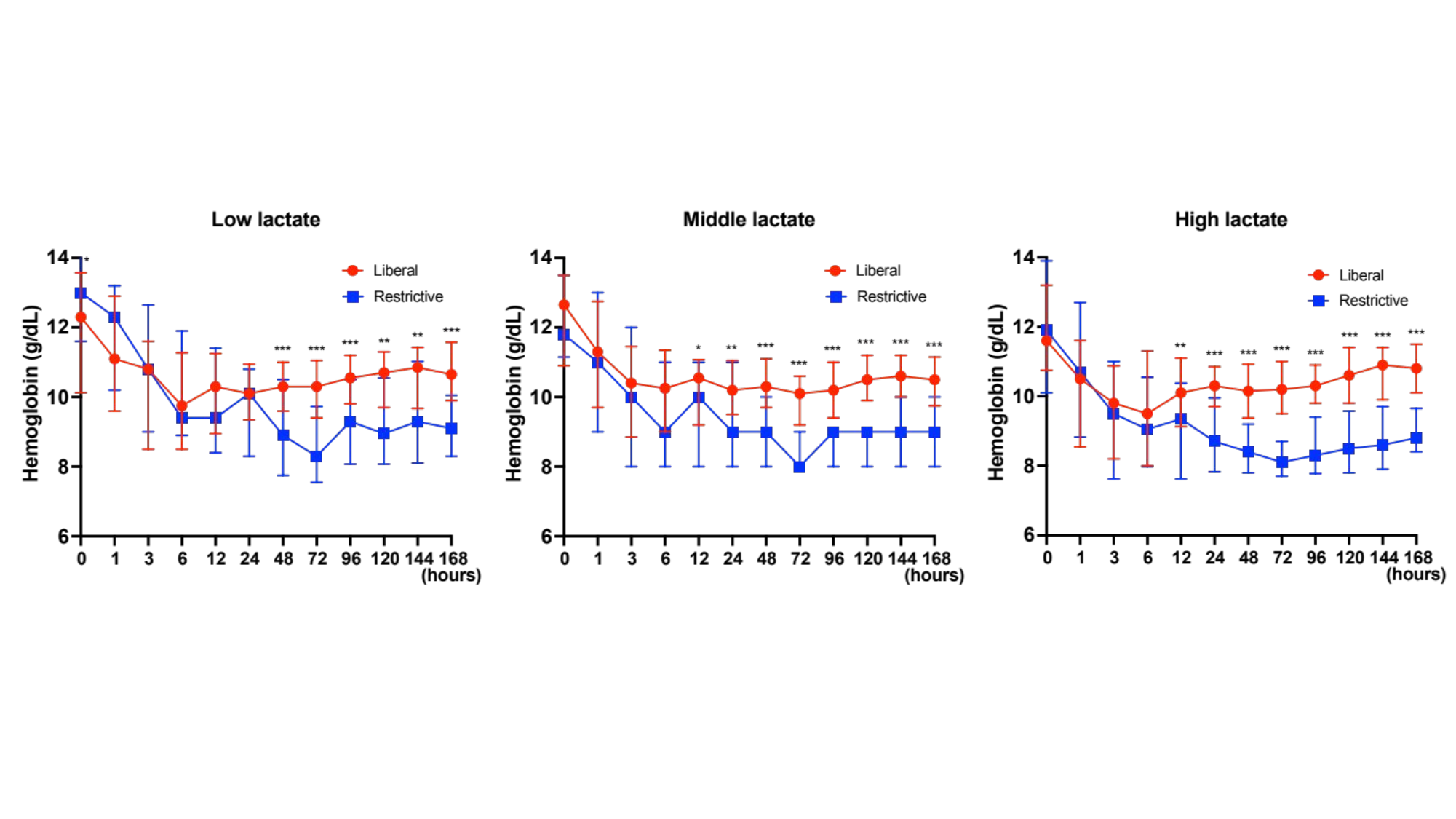

Supplement: Supplementary file 1 — Additional file 1. Trajectory of hemoglobin concentrations in the restrictive versus liberal strategy groups according to initial lactate levels. Data are expressed as medians with interquartile ranges. Indicator of significance was reported by adjusted P value as *p < 0.05, **p < 0.01, and ***p < 0.001 in figure. [file 13017_2023_530_MOESM1_ESM.pptx]

## Slide 1
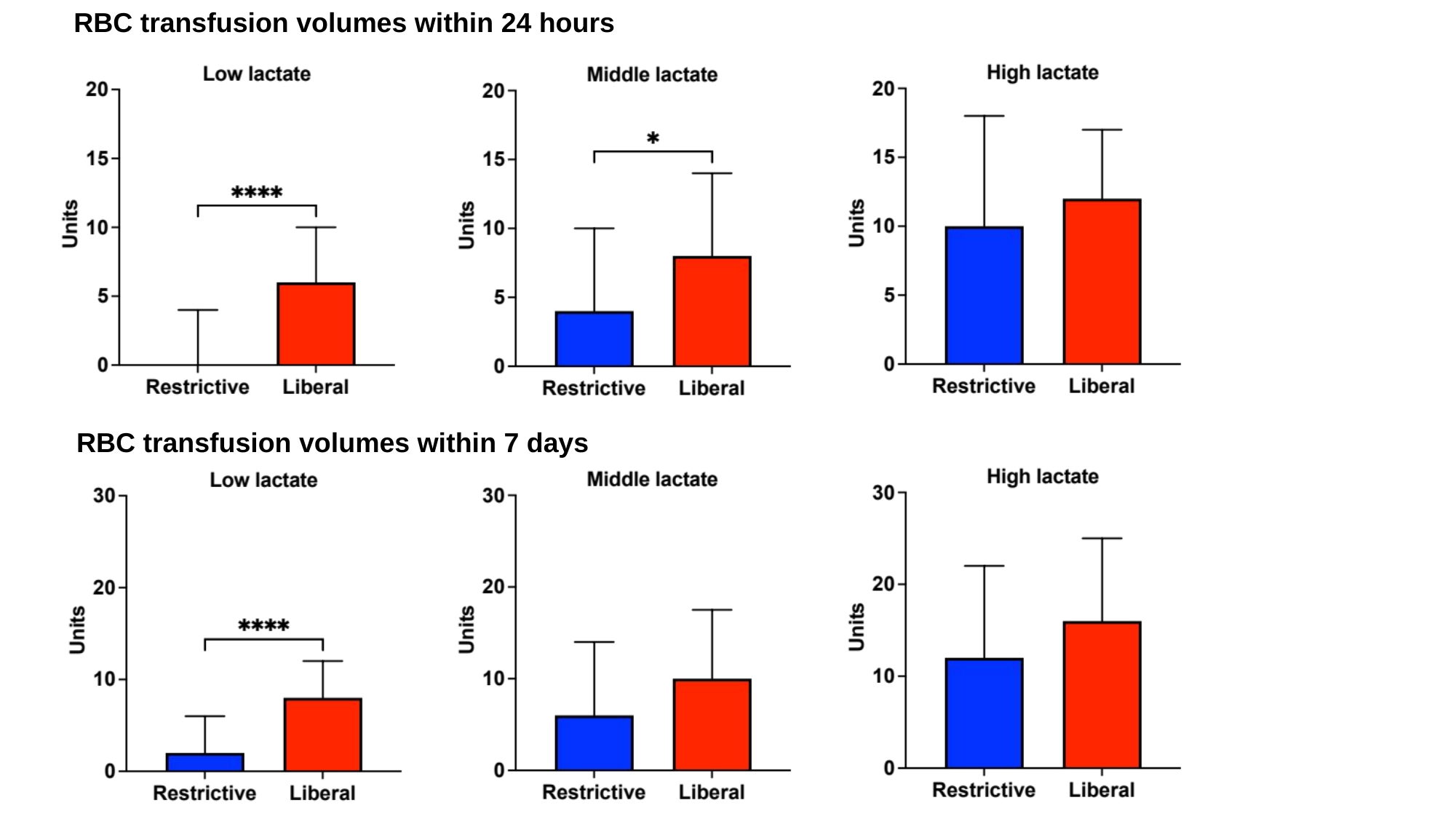

RBC transfusion volumes within 24 hours
RBC transfusion volumes within 7 days

Supplement: Supplementary file 2 — Additional file 2. Cumulative RBC transfusion volumes comparing between restrictive and liberal strategy based on initial lactate levels within 24 h and 7 days. Data are expressed as medians with interquartile ranges. Indicator of significance was reported by adjusted P value as *p < 0.05 and ****p < 0.0001 in figure. RBC: red blood cell. [file 13017_2023_530_MOESM2_ESM.pptx]

## Slide 1
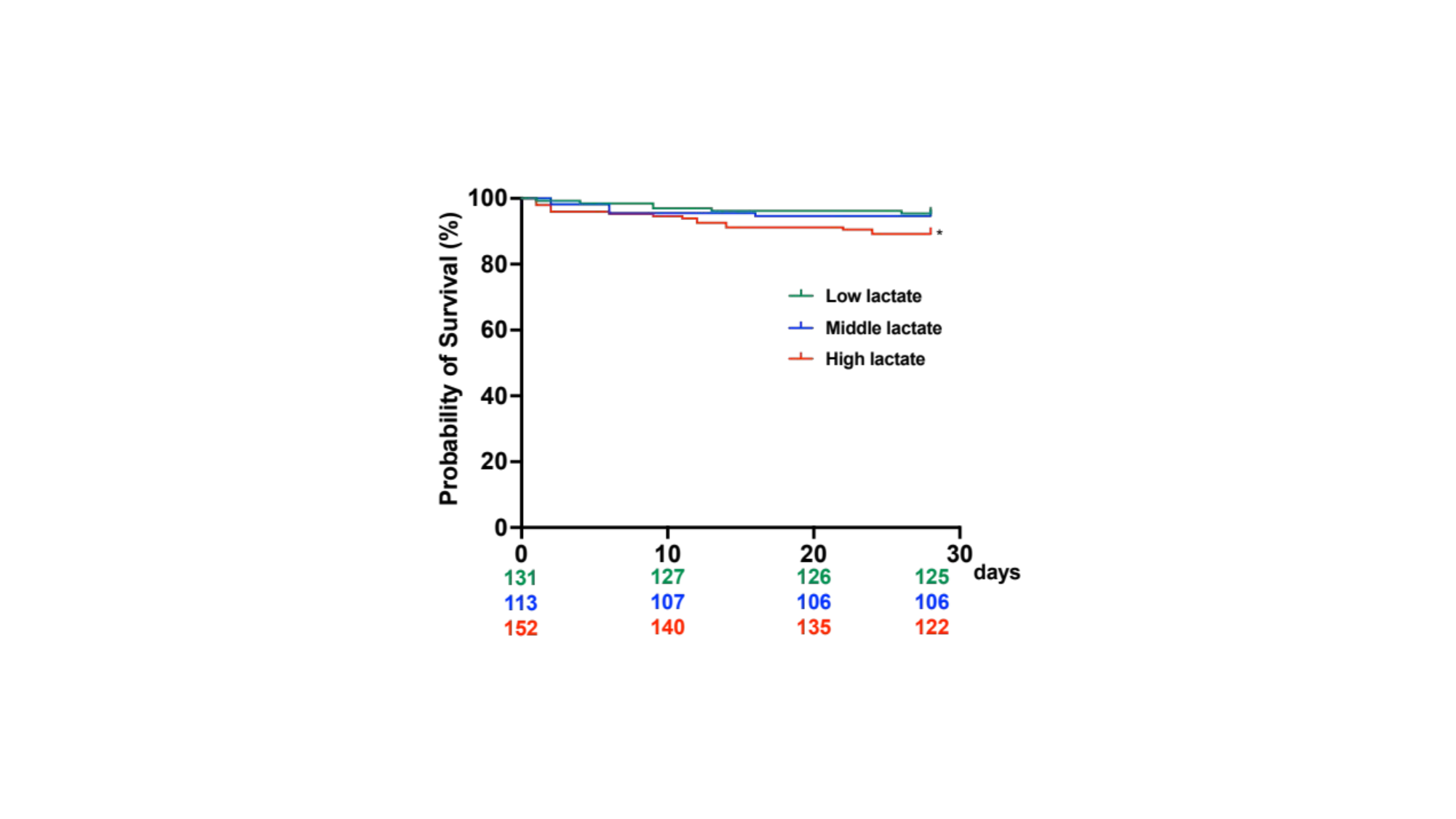

Supplement: Supplementary file 3 — Additional file 3. Kaplan–Meier survival analysis for 28-day survival comparing between the low, middle, and high lactate groups. Colored numbers at the bottom of the plot illustrate the number of patients at risk in the respective strategy at specified timepoints. *p = 0.041 compared with low the lactate group. [file 13017_2023_530_MOESM3_ESM.pptx]

## Slide 1
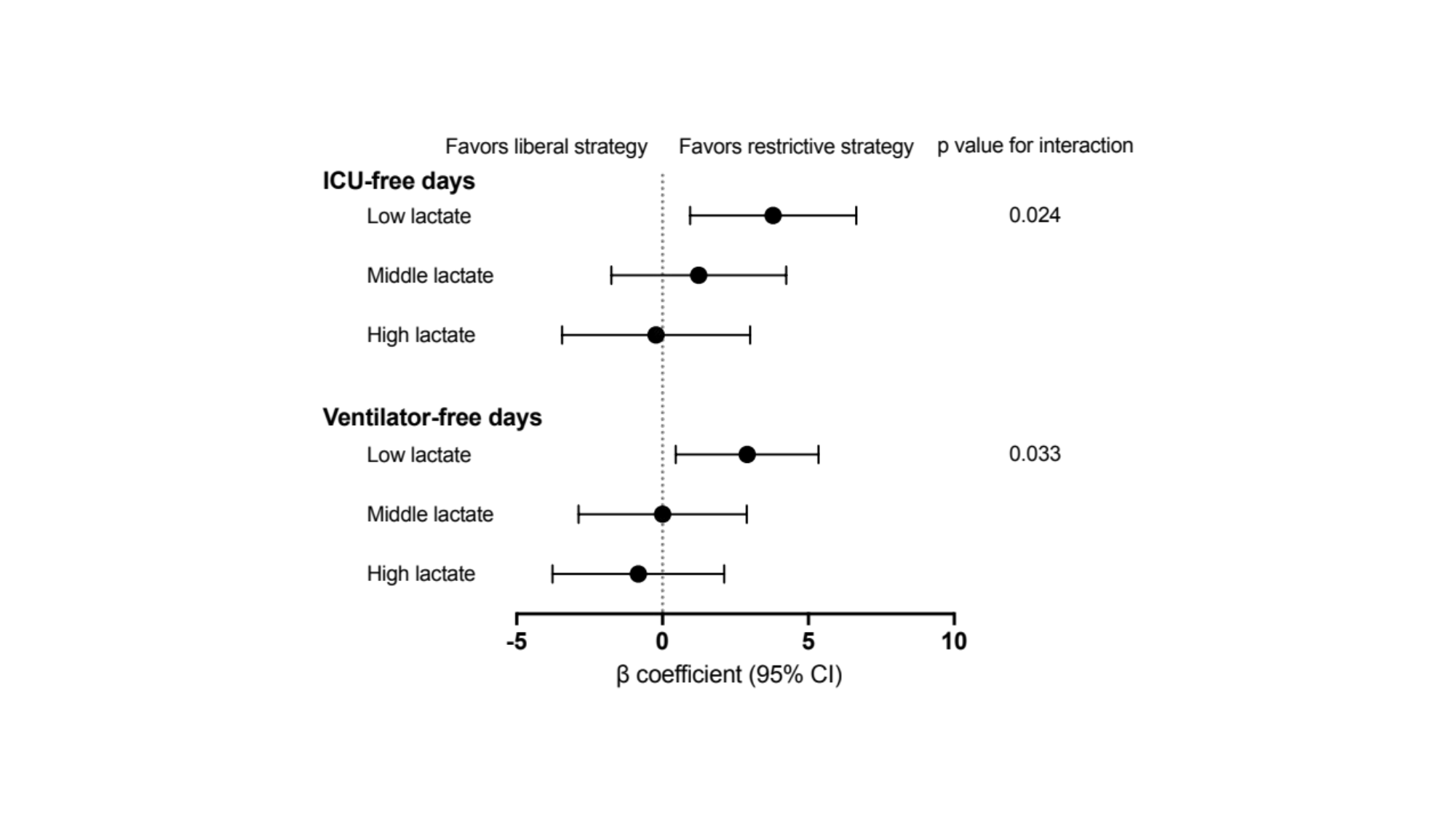

Supplement: Supplementary file 5 — Additional file 5. Multiple linear regression analyses to estimate adjusted effects of restrictive vs. liberal RBC transfusion strategy on ICU-free and ventilator-free days after excluding patients with severe traumatic brain injury, according to the initial lactate levels. Analyses were conducted for each lactate level category and were adjusted for factors including age, sex, systolic blood pressure, injury severity score, initial hemoglobin levels, and the need for major hemostatic interventions. RBC red blood cells, ICU intensive care unit, CI confidence intervals. [file 13017_2023_530_MOESM5_ESM.pptx]
