# Supplementary material for: Association of initial lactate levels and red blood cell transfusion strategy with outcomes after severe trauma: a post hoc analysis of the RESTRIC trial
Source: World J Emerg Surg. 2024 Jan 2;19:1. doi: 10.1186/s13017-023-00530-7 (PMC10763143; doi:10.1186/s13017-023-00530-7)
Supplement: Supplementary file 4 — Additional file 4. Multiple logistic regression analyses to estimate adjusted effects of restrictive RBC transfusion strategy on any organ failure and any complications, according to the initial lactate levels, setting liberal strategy as a reference. [file 13017_2023_530_MOESM4_ESM.docx]

**Additional file 4.** Multiple logistic regression analyses to estimate adjusted effects of restrictive RBC transfusion strategy on any organ failure and any complications, according to the initial lactate levels, setting liberal strategy as a reference.

|  | Low lactate | |  | Middle lactate | |  | High lactate | |
| --- | --- | --- | --- | --- | --- | --- | --- | --- |
|  | adjusted OR | 95% CI |  | adjusted OR | 95% CI |  | adjusted OR | 95% CI |
| Any organ failure | 0.82 | 0.17 to 4.04 |  | 3.37 | 0.76 to 21.38 |  | 0.75 | 0.27 to 2.01 |
| Any complications | 1.45 | 0.46 to 4.74 |  | 0.77 | 0.27 to 2.19 |  | 0.79 | 0.32 to 1.92 |

Analyses were conducted for each lactate level category and were adjusted for factors including age, sex, systolic blood pressure, the presence of a severe head injury (defined as an Abbreviated Injury Scale of 4 or 5 for the head), Injury Severity Score, initial hemoglobin levels, and the need for major hemostatic interventions.

RBC: red blood cells, ICU: intensive care unit, OR: odds ratio, CI: confidence intervals
